# CuDDI: A CUDA-Based Application for Extracting Drug-Drug Interaction Related Substance Terms from PubMed Literature

**DOI:** 10.3390/molecules24061081

**Published:** 2019-03-19

**Authors:** Yin Lu, Aditya Chandra Vothgod Ramachandra, Minh Pham, Yi-Cheng Tu, Feng Cheng

**Affiliations:** 1 Department of Pharmaceutical Science, College of Pharmacy, University of South Florida, Tampa, FL 33612, USA; yinlu86@gmail.com; 2 Department of Computer Science and Engineering, University of South Florida, Tampa, FL 33612, USA; adityachandr@mail.usf.edu; 3 Department of Mathematics and Statistics, University of South Florida, Tampa, FL 33620, USA; minhpham@mail.usf.edu; 4 Department of Epidemiology and Biostatistics, College of Public Health, University of South Florida, Tampa, FL 33612, USA

**Keywords:** drug-drug interaction, PubMed, CUDA, random sampling, GPU, Substance, term, mechanism, parallel computing

## Abstract

Drug-drug interaction (DDI) is becoming a serious issue in clinical pharmacy as the use of multiple medications is more common. The PubMed database is one of the biggest literature resources for DDI studies. It contains over 150,000 journal articles related to DDI and is still expanding at a rapid pace. The extraction of DDI-related information, including compounds and proteins from PubMed, is an essential step for DDI research. In this paper, we introduce a tool, CuDDI (compute unified device architecture-based DDI searching), for identification of DDI-related terms (including compounds and proteins) from PubMed. There are three modules in this application, including the automatic retrieval of substances from PubMed, the identification of DDI-related terms, and the display of relationship of DDI-related terms. For DDI term identification, a speedup of 30–105 times was observed for the compute unified device architecture (CUDA)-based version compared with the implementation with a CPU-based Python version. CuDDI can be used to discover DDI-related terms and relationships of these terms, which has the potential to help clinicians and pharmacists better understand the mechanism of DDIs. CuDDI is available at: https://github.com/chengusf/CuDDI.

## 1. Introduction 

A drug-drug interaction (DDI) occurs when the pharmacologic effect of a given drug is altered by the action of another drug, leading to unpredictable clinical effects [1]. DDIs may make the drug less effective, delay drug absorption, or cause unexpected harmful side effects [2]. In 2007, DDIs caused approximately 0.054% of emergency room visits, 0.57% of hospital admissions, and 0.12% of rehospitalizations in the United States [3]. Polypharmacy, the concurrent use of multiple medications, is an important factor for increasing the risk of DDIs [3]. Therefore, detecting DDIs is of great interest to the pharmaceutical industry, drug regulatory agencies, healthcare professionals and patients [4]. DDIs are frequently reported in clinical and scientific journals [5,6,7]. The PubMed, developed by the US National Library of Medicine, contains over 29 million entries from more than 5600 journals, with 2000–4000 new references being added daily. Searching PubMed for journal articles related to DDI produces over 150,000 results. PubMed records have a well-defined structure which includes the title of the journal article, author information, journal information, publication type, language, abstract, MeSH (medical subject headings) terms, and substances. Among them, substances and MeSH terms are manually indexed by specialized personnel to provide important information about the whole article. Substances include chemicals and chemical-reaction-related enzymes mentioned in the paper. To identify DDI information from PubMed literature, we have developed a random sampling-based statistical algorithm using substances [8,9]. However, the random sampling steps in the second step are time-consuming for a large number of articles. The main issue is that each of the sampling steps is processed one by one in a single thread. Since the sampling steps are independent, parallel processing is obviously the strategy to follow to boost performance. Modern graphics processing units (GPUs), with their extremely high level of parallelism and large memory bandwidth, are therefore a desirable platform for implementing such computations. Programming frameworks such as Nvidia’s compute unified device architecture (CUDA) have allowed convenient software development on GPUs. In this paper, we introduce a CUDA-based application, CuDDI (CUDA-based DDI searching) for the identification of DDI-related terms (including compounds and proteins) from PubMed. There are three steps in this algorithm, including the retrieval of substances from PubMed literature, the identification of DDI-related terms, and the display of relationship of DDI-related terms. A CUDA-based implementation of the second step of this algorithm can dramatically speed up the processing using parallel computing.

## 2. Results

### 2.1. Input and Output of CuDDI. 

The input of CuDDI is a drug name (for example, Simvastatin) that is stored in the file “drugNameList_original.txt”. A parameter file (as shown in Table 1) includes a list of parameters that are used for data collection and p-value calculation in CuDDI. The parameter sampleTimes is defined as random sampling times. The cutoff specifies the co-occurrence of the candidate compounds/proteins and the queried drug in the same paper. Z_score, used for *p*-value calculation, indicates how many standard deviations the co-occurrence is from the mean. The endDate is the end date of the search for the publications in PubMed. The endDate argument must be in the format of YYYY/MM/DD. 

**Figure 1:**
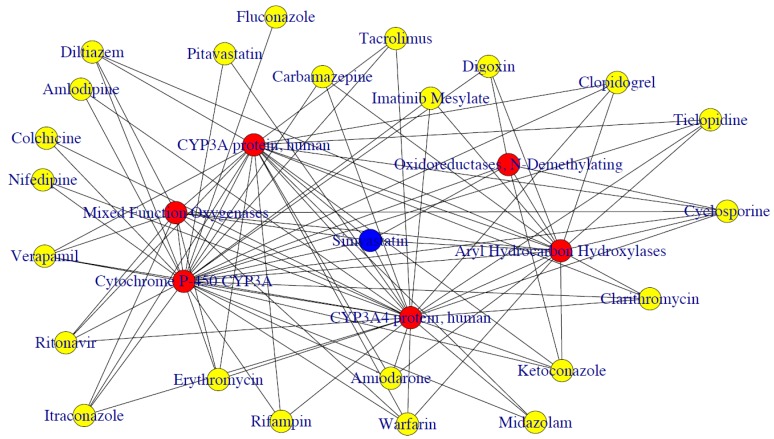
The social network of the proteins and FDA approved drugs identified from CuDDI.

The output of CuDDI is the compound/protein terms and the corresponding frequency *p*-values and adjusted *p*-values. As shown in Table 2, 33 FDA approved drugs were identified from PubMed with the frequency >3 and adjusted *p*-value <0.05 for simvastatin. Among 33 FDA approved drugs, 26 drugs were proven to interact with simvastatin (accuracy = 78.8%). The results showed CuDDI could correctly identify most of the DDI-related terms from PubMed. It was also shown that CYP450 enzymes, especially CYP3A4, were identified, which indicates simvastatin is involved in pharmacodynamic DDIs [8]. The social network of these terms was plotted by CuDDI to show the relationship among terms (Figure 1). The connections between two terms in the figure indicate that these terms are in the same paper.

### 2.2. The Performance of CuDDI Comparing with a CPU-Based Python Version 

We applied both the CPU-based Python-based and CUDA-based codes to investigate simvastatin using the same parameters (frequency threshold >3, adjusted *p*-value threshold < 0.05 and random sampling times = 1000). As shown in Table S1, the frequency and ranking of the DDI-related compounds and proteins were the same for these two codes. There was a slight difference in some adjusted *p*-values because of the random sampling step. Different articles in the non-DDI group were randomly sampled in every run.

**Figure 2:**
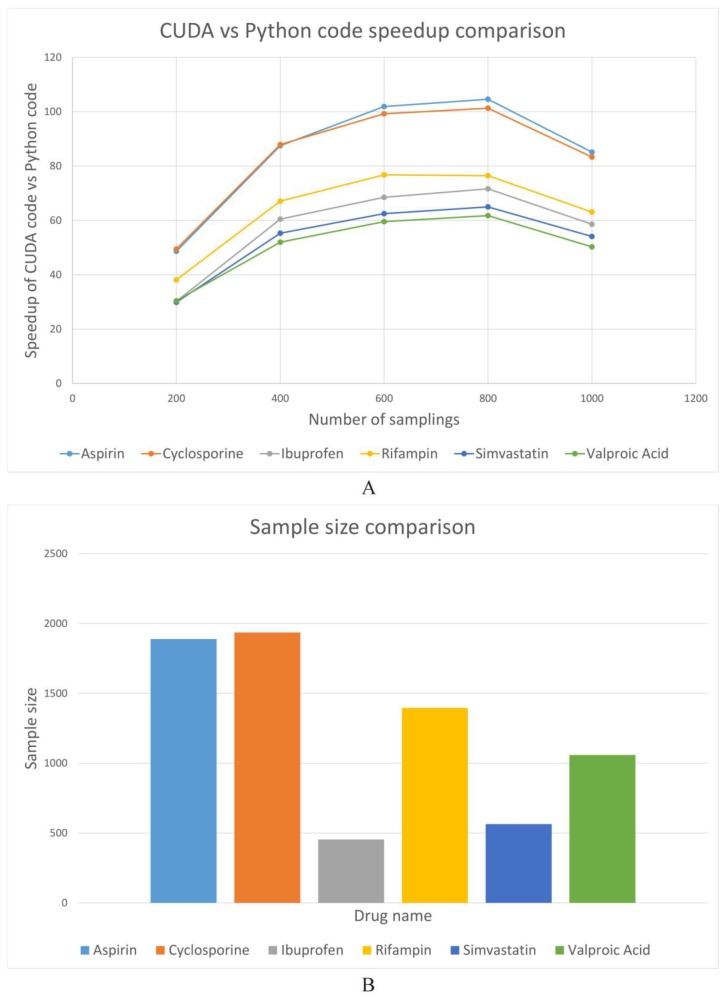
(**A**) The comparison of the speedup of the compute unified device architecture (CUDA) vs. Python code for different threads across six drugs. (**B**) The comparison of sample size for six drugs.

Six drugs, including aspirin, cyclosporine, ibuprofen, rifampin, simvastatin and valproic acid, were selected to test the code with varying times of samplings from 200 to 1000. The threshold of frequency and *p*-value were set to be 1 and 0.1. The CPU was the POWER8NVL (raw) with the clock at 2061 MHz and the GPU was the Tesla P100-SXM2 with a memory of 16280 MiB. The running time of CUDA code (GPU version) and Python code (CPU version) is shown in Table S2. The speedup comparison between the Python code of six drugs is calculated and displayed in Figure 2A. The speedup is defined by the running time of Python code divided by running time of the CUDA code. We can see that the speedup increased with the increase of sampling times when it was lower than 800. However, the speedup decreased when sampling times reached 1000. This could be caused by two reasons: (1) If the sample size is larger than 1024, some threads will randomly sample more than one record; (2) The warp level divergence is caused by variations in the number of keywords in randomly picked records. Figure 2B shows the sample size for six drugs, which are 1889, 1936, 454, 1396, 564 and 1059 for aspirin, cyclosporine, ibuprofen, rifampin, simvastatin and valproic acid, respectively. Clearly, the CUDA code outperformed the Python code by a great margin and the speedup was at least 30 fold. The largest speedup is 105 times for the case of aspirin, under the number of sampling of 800. 

## 3. Conclusion and Discussion

CuDDI is a CUDA-based application for the identification of DDI-related terms (including compounds and proteins) from PubMed. Although some public databases (such as drugbank or drugs.com) have provided information about common clinically important DDIs, CuDDI can explore DDIs that were only reported in the research paper. In addition, CuDDI can be used to explore the possible mechanisms of DDIs by analyzing relationships of DDI terms, which has the potential to help clinicians and pharmacists better understand DDIs. 

CUDA on the general purpose (GP) GPU platform can be designed to exploit data level parallelism very effectively by fully utilizing the power of GPUs. The CuDDI is a good example for improving performance with CUDA techniques. As the problem of random sampling is highly parallelizable, all the random samplings can be performed in parallel. Our solution is perfect to leverage on the Nvidia GPUs with the CUDA architecture, where every block of threads executes a random sampling in parallel. Although Python code did not take a much longer time for six drugs in our paper, for 1000 steps of sampling, if we run a web service that may accept many queries or many steps of sampling in a short period of time, running one query in a few seconds would be a problem. As the PubMed database continues to grow, this CUDA-based technique of identifying DDIs proves to be very efficient, scalable and beneficial.

There are some limitations to this approach. First, currently, CuDDI is designed specifically for investigating DDIs from research articles in PubMed because the substance terms are only available in the PubMed database. However, the tool can be modified to investigate DDIs in other social media (such as Yahoo Answers, Facebook or Twitter) by adding steps of keyword extraction and normalization. Second, some low-frequency DDI terms are excluded to avoid the noise. However, these kinds of DDIs can be detected in the future with new references (2000–4000 papers per day) being added in PubMed. Third, some important proteins, such as transporters, are not included in substances in PubMed. Substances generally include chemicals and chemical reaction-related enzymes. In the future, we plan to consider MeSH terms in CuDDI. Many transporter proteins have been included in the newest version of the MeSH terms. Fourth, our approach requires CUDA capable Nvidia GPUs to exploit the power of data level parallelism using the GPGPU platform. Nevertheless, with the moderate price of powerful Nvidia GPUs and the standard PCI-E (Peripheral Component Interconnect Express) link required to support GPUs from the host machine, this will not become a major obstacle for most users. In conclusion, as the volume of PubMed records increases, CuDDI will become more efficient and accurate for drug interaction research.

## 4. Methods

**Figure 3:**
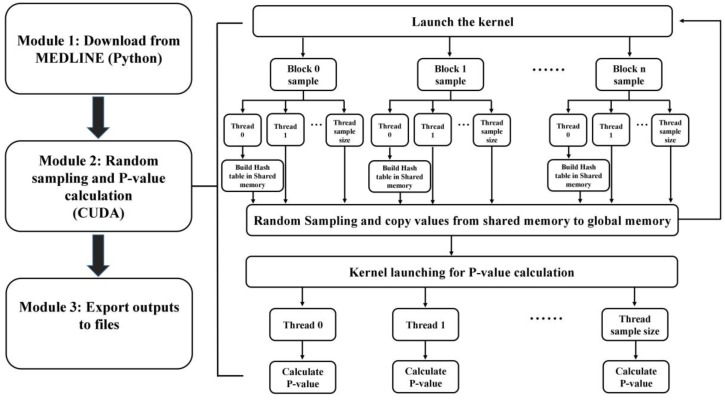
The flowchart of the process in CuDDI.

As shown in Figure 3, the process of the CuDDI application was classified into three modules: Downloading DDI related substances, random sampling *p*-value calculation, and displaying the relationship among the significant DDI-related terms. The input of CuDDI was a drug name. The first module involved downloading DDI related information from PubMed, which could be accessed with Biopython [10]. All PubMed records containing the queried drug were downloaded. Substance terms of the retrieved records were then extracted. The articles were separated into two groups: Articles containing drug interactions (Group A) and articles without drug interactions (Group B). The compounds and proteins in the substances field of articles in Group A were selected as the candidate terms. 

In Module 2, these terms are accessed by the CUDA C program [11] to perform a random sampling-based statistical analysis to compare the co-occurrence of terms with the drug of interest in the articles of Group A and those of Group B. The *p*-value for each candidate was calculated. 

The random sampling step was highly parallelizable, each sample could be collected and processed in parallel to utilize the power of the GPU effectively. Based on empirical evaluations, to achieve the highest utilization of global memory bandwidth, the CUDA kernel was launched with 256 or fewer blocks and each block consisted of n threads, where n was the sample size. In the kernel, each block corresponding to one sampling and every thread would randomly sample a record, which was already sorted based on the number of keywords. Sorting was essential to reduce warp level divergence, which was caused by the different numbers of keywords that would be processed by neighboring threads within a warp. Before sampling, a hash table of the keywords with interaction was built in shared memory by the first thread within each block. The shared memory was part of the processor chip and the hash table in the shared memory could be accessed with lower latency and a much higher bandwidth than the global memory by all the threads within the same block. During sampling, every thread would process one PubMed record that was picked up based on generated random numbers. The keywords from the corresponding record were then extracted.. All the keywords were searched through the hash table and the values were incremented if a key is found. To make way for the *p*-value calculation, shared memory values were copied to the global memory after the sampling. The calculation of the *p*-value was a simple reduction, every thread would compute the mean, standard deviation and Z-score for one record. The normal distribution of the Z-score was populated as the *p*-value, the lower the *p*-value the higher the probability of a DDI. An adjusted *p*-value was calculated subsequently to control for the false discovery rate (FDR) by using the Benjamini–Hochberg approach [12].

In Module 3, the relationship among terms with significant adjusted *p*-values was shown by social network analysis. A weighted social network was constructed with nodes representing substances and the edges representing interactions between two connected nodes. The term networks were plotted using igraph packages in R (https://www.r-project.org). Detailed information about the algorithm can be found in our previous papers [8].

## Figures and Tables

**Table 1:** The parameters for CuDDI.

Parameters	Value
Sample Times	1000
cutoff	4
*p-*value	0.05
Z-score	1.645
end date	2015/12/31

**Table 2:** The proteins and Food and Drug Administration (FDA) approved drugs identified from CuDDI for searching simvastatin with the frequency >3 and adjusted *p*-value < 0.05.

Terms	Frequency	*p*-Value	DDI with Simvastatin *
**FDA Approved Drugs**			
Cyclosporine	35	0	Y
Warfarin	23	0	Y
Diltiazem	19	0	Y
Ticlopidine	18	0	N
Clopidogrel	18	0	N
Clarithromycin	17	0	Y
Amiodarone	17	0	Y
Itraconazole	15	0	Y
Aspirin	15	0.023	Y
Verapamil	12	0	Y
Rifampin	11	0	Y
Ketoconazole	11	0	Y
Amlodipine	9	0	Y
pitavastatin	9	0.0083	Y
Ritonavir	7	0	Y
Digoxin	7	0	Y
Midazolam	7	0	N
Erythromycin	6	0	Y
Imatinib Mesylate	5	0	Y
Colchicine	5	0	Y
Tacrolimus	5	0	Y
Nifedipine	5	0.000083	Y
Sirolimus	5	0.0016	Y
Lisinopril	5	0.0032	N
Atenolol	5	0.0053	N
Ramipril	5	0.026	N
Nelfinavir	4	0	Y
nefazodone	4	0	Y
Ranolazine	4	0	Y
Troglitazone	4	0	Y
Carbamazepine	4	0	Y
Fluconazole	4	0	Y
Sitagliptin Phosphate	4	0.0000052	N
**Proteins**			
Cytochrome P-450 CYP3A	93	0	
CYP3A4 protein, human	57	0	
CYP3A protein, human	34	0	
Mixed Function Oxygenases	16	0	
Aryl Hydrocarbon Hydroxylases	16	0.0024	
Oxidoreductases, *N*-Demethylating	4	0.0018	

* Drug interaction information from Drug Interaction Checker (http://www.drugs.com/drug_interactions.html) was used as the gold standard data for validation.

## Supplementary Materials

Supplementary Materials are available online. Table S1: The proteins and FDA approved drugs identified from CuDDI for searching simvastatin by CPU-based python based and CUDA-based code with the same parameters (frequency > 3, adjusted *p*-value < 0.05, and random sampling times = 1000); Table S2: The running time of the CUDA code (GPU version) and Python code (CPU version).
